# Book Review: Occupational Health for Health Care Professionals

**Published:** 2008-01-01

**Authors:** D.M. Thuraiappah

**Affiliations:** 1 Assoc. Prof. (Adj.), Malaysia

This compendium of essays by 30 authors is a contribution to the Malaysian ever growing storehouse of medical publications. It is a worthwhile project for the Malaysian Medical Association to have undertaken to publish this long awaited book, because the content of the book involves the care of its own members. The health of the healthcare providers is often taken for granted while carrying out their duties of a doctor. They forget their own health and they expose themselves to the risk of disease every day of their lives.

This book, with twenty-two chapters, covers in detail the occupational concerns of health care professionals. The chapters outline the common pitfalls in the healthcare system into which the professionals may fall into. All health care facilities are high risk venues for which not sufficient preventive systems are in place. The various risk factors are highlighted by the different authors both from the point of view of the professional and the patient. In support of preventive efforts the authors refer to the various statutory requirements in place. In spite of the provisions, the authors cite many instances of diseases and disasters the professional suffer from and are exposed to daily.

This book will be of use both not only to the student of occupational health but also to every healthcare professional. It raises the awareness of personal protection and prevention since the chance of disaster awaits every morning. The dictum of “Physician, heal thyself” may come too late if this book does not evoke caution every day. It is well written with cases documenting poor infrastructure requirements to carry out their duties in a safe and efficient manner. References are well documented by all the authors to inspire further work in this area. Associate Professor Dr Jayakumar comes from the backgrounds of both academic and corporate sectors and therefore contributes his wealth of knowledge and experience while Associate Professor Dr. Ratneswari coming from a teaching background carries off the vast academic references with aplomb. The book is well compiled and the authors must be congratulated for bringing out this timely book.

